# Intervention strategies for cancer-related sarcopenia: a scoping review

**DOI:** 10.3389/fnut.2025.1666547

**Published:** 2025-12-18

**Authors:** Lei Li, Haiyan Zhu, Jiangxia Chen, Lingchang Shan, Jiamin Xu

**Affiliations:** 1 Department of Gastrointestinal Surgery, Shaoxing People's Hospital, The First Hospital of Shaoxing University, Shaoxing, Zhejiang, China; 2 Department of Nursing, Shaoxing People's Hospital, The First Hospital of Shaoxing University, Shaoxing, Zhejiang, China; 3 Department of General Practice, Shaoxing People's Hospital, The First Hospital of Shaoxing University, Shaoxing, Zhejiang, China; 4 School of Medicine, Shaoxing University, Shaoxing, Zhejiang, China

**Keywords:** cancer, sarcopenia, intervention strategies, rehabilitation, randomized controlled trial (RCT)

## Abstract

**Objective:**

This article systematically reviewed intervention strategies for cancer-related sarcopenia (CRS), providing evidence for researchers to develop targeted treatments.

**Methods:**

PubMed, Embase, Web of Science, and the Cochrane Library were searched for studies between 2015 and 2025, followed by literature screening and content analysis.

**Results:**

A total of 3,566 articles were initially identified, and 18 randomized controlled trials (published between 2016 and 2025; sample sizes ranging from 15 to 232) were ultimately included. CRS interventions were categorized into four types: nutritional, exercise, pharmacological, and multidisciplinary.

**Conclusion:**

A CRS intervention needs an integrated approach that combines nutrition, exercise, pharmacology, and a multidisciplinary team (MDT) to improve patients’ functional outcomes and quality of life. Future research should focus on precision approaches and translational medicine.

## 1 Introduction

According to the 2022 Global Cancer Statistics, the total number of cancer cases is projected to increase to 35.3 million by 2050, indicating a severe global cancer challenge (1). On 2 February 2024, the International Agency for Research on Cancer (IARC) again highlighted the escalating global cancer burden in its latest report, underscoring the urgent need for worldwide attention (2). CRS is one of the key issues emphasized. It is a syndrome characterized by progressive decline in skeletal muscle mass, strength, and function in cancer patients, caused either by the tumor itself or anticancer treatments (3). Its incidence varies significantly across cancer types, reaching 60–80% in patients with pancreatic, gastric, and lung cancers, while remaining relatively lower (20–30%) in those with breast and prostate cancers (4, 5). Unlike age-related sarcopenia, CRS progresses more rapidly, increasing the risk of postoperative complications, reducing chemotherapy tolerance, significantly impairing quality of life, and shortening survival. Annual medical expenditures for CRS patients were 1.5 to 2 times higher than those for non-CRS patients, primarily due to increased hospitalizations, management of complications, and rehabilitation treatments (6). Therefore, early prevention and intervention for CRS are urgent global public health challenges that need to be addressed.

The factors affecting CRS are numerous. Tumor-related factors include inflammatory cytokines secreted by tumors, such as interleukin-6 (IL-6) and tumor necrosis factor-α (TNF-α), which activate the ubiquitin–proteasome system (UPS), accelerating muscle protein degradation. In addition, the Warburg effect leads to systemic energy depletion and disrupted glucose and lipid metabolism in muscle tissue (7). Treatment-related factors include chemotherapy drugs such as platinum-based agents and paclitaxel, which directly impair mitochondrial function and inhibit muscle regeneration. Radiation therapy induces local inflammatory reactions and oxidative stress, resulting in muscle fibrosis. Prolonged bed rest after surgery further accelerates muscle atrophy (8, 9). Patient-related factors include the hypermetabolic state caused by tumors, which leads to insufficient protein intake, particularly a deficiency in branched-chain amino acids (BCAAs). Pain, fatigue, and psychological depression further reduce physical activity. In addition, insulin resistance and dysregulation of the growth hormone (GH)/insulin-like growth factor I (IGF-1) axis suppress muscle synthesis (10). Given these influencing factors, implementing targeted intervention measures to prevent and treat CRS is a question worthy of in-depth research.

At present, insufficient attention is paid to sarcopenia in the treatment of cancer patients. Therefore, we believe that actively implementing interventions for CRS in clinical practice is closely associated with improved treatment outcomes, enhanced patient prognosis, better overall quality of life, and higher healthcare quality.

To address this critical issue, this article adopts a literature review approach to explore interventions for CRS from multiple dimensions, including nutrition, exercise, medication, and multidisciplinary management, with the aim of providing a reference for clinical practice and research.

## 2 Methods

We conducted a scoping review adhering to the Preferred Reporting Items for Systematic Reviews and Meta-Analyses (PRISMA) Scoping Review extension (PRISMA-SCR) checklist.

### 2.1 Search strategy

Research question: What are the intervention strategies for CRS?

To address the aforementioned research question, a systematic literature search was conducted in February 2025. A combination of Medical Subject Headings (MeSH) and free-text terms was used to systematically retrieve articles from PubMed, Embase, Web of Science, and the Cochrane Library (accessed via the Ovid research platform). The MeSH terms included “Neoplasms,” “Sarcopenia,” and “randomized controlled trial,” and the search was limited to publications from 2015 to 2025.

### 2.2 Inclusion and exclusion criteria for literature

#### 2.2.1 Inclusion criteria

Studies were considered eligible if they met the following criteria: (1) participants were aged≥18 years; (2) patients had a pathological diagnosis of cancer; and (3) studies were published in English.

#### 2.2.2 Exclusion criteria

Studies were excluded if they met any of the following conditions: (1) the literature data were incomplete; (2) the literature quality was poor; or (3) the literature type consisted of preliminary experiments or similar reports.

### 2.3 Literature screening

The retrieved literature was imported into EndNote, and the titles and abstracts were preliminarily reviewed to exclude studies clearly irrelevant to this research. Two researchers independently evaluated the full texts of the remaining articles and cross-checked their selections. If discrepancies arose, they were resolved through discussion or, if necessary, adjudicated by a third researcher, resulting in the final set of included studies.

### 2.4 Data extraction

Data extraction was performed independently by two researchers. The extracted information included author details, year of publication, country, sample volume, type of study, intervention measures, and results. The extracted data were then compiled and cross-verified by the researchers.

### 2.5 Quality assessment of the literature

The included randomized controlled trials (RCTs) were evaluated for quality using Version 2 of the Cochrane Risk of Bias tool for randomized trials (RoB2) (11).

## 3 Results

### 3.1 Results of literature search

**Figure 1:**
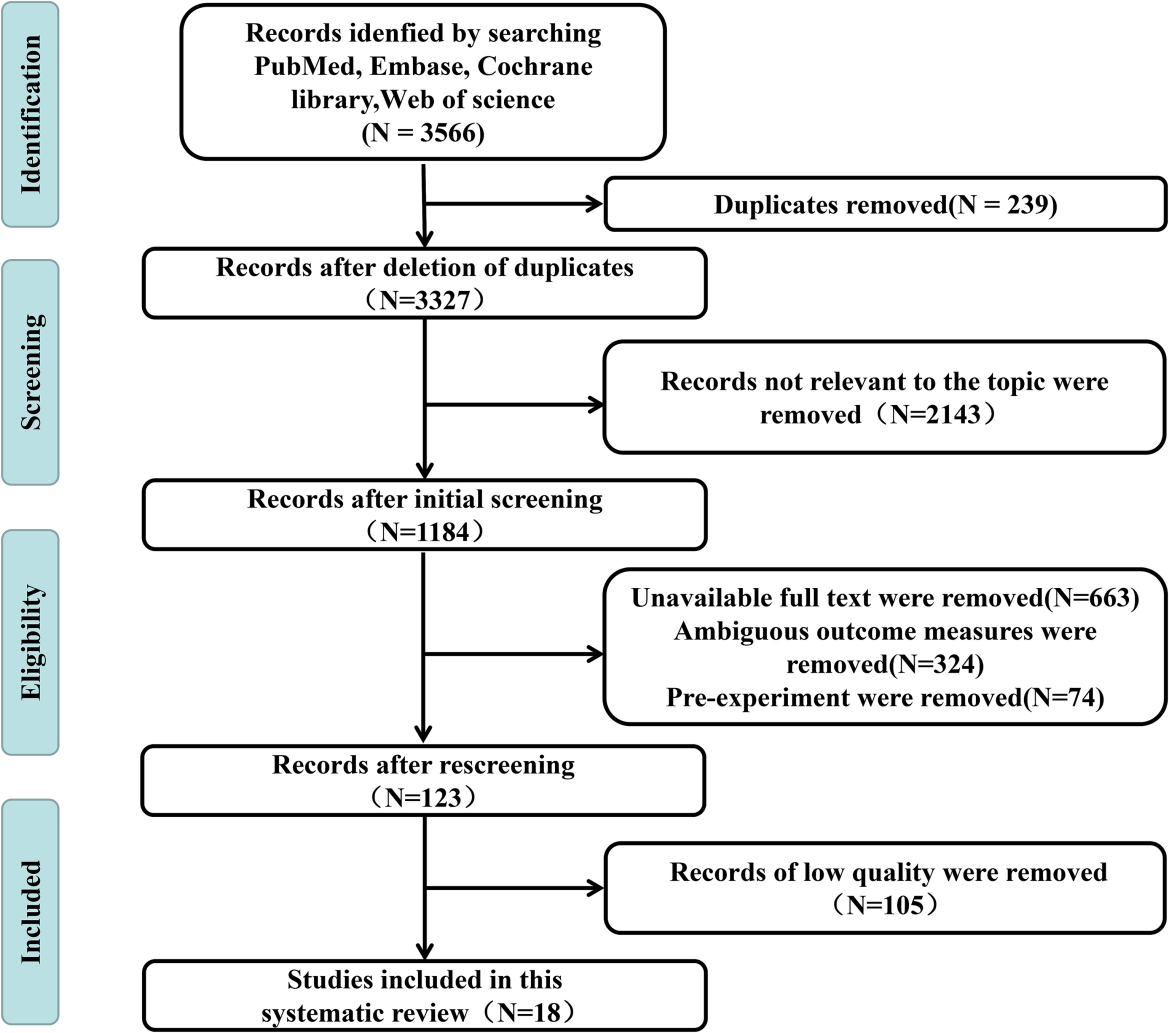
PubMed, Embase, Web of Science, and the Cochrane Library were systematically searched for research questions between 2015 and 2025. The included studies were identified through literature screening. A total of 18 studies were included, all of which were randomized controlled trials.

Through systematic retrieval, we obtained a total of 3,566 articles, including 340 from PubMed, 471 from Embase, 823 from Web of Science, and 1,932 from the Cochrane Library. After stepwise screening, 18 articles were ultimately included for analysis. The detailed literature screening flowchart is presented in Figure 1.

### 3.2 Basic characteristics of the included literature

Eighteen studies published between 2016 and 2025 were included, with sample sizes ranging from 15 to 232 participants. The majority of the studies were conducted in the USA (*n* = 7, 38.9%), with the remaining studies from the UK (*n* = 3, 16.7%) and South Korea (*n* = 2, 16.7%), and one study each from Spain, China, Lithuania, the Netherlands, Italy, Saudi Arabia, and Egypt. The basic characteristics of the included studies are presented in Table 1. A summary table comparing intervention efficacy across categories is presented in Table 2, and the results of the risk of bias assessment table for randomized controlled trials are presented in Table 3.

**Table 1:** Basic characteristics of the included literature.

Author	Published year	Country	Sample volume	Type of study	Type of cancer	Intervention	Control	Results
Herrera-Martínez et al. (12)	2023	Spain	46	RCT	Multiple types of cancers	Hypercaloric and hyperproteic leucine-enriched OS	Whey protein-based hyperproteic oral supplements	Extracellular mass tended to increase in patients who received the leucine-enriched formula. Functionality (evaluated through the stand-up test) improved in both groups (*p*<0.001). Prealbumin, transferrin levels, and superficial adipose tissue increased in the control group (*p*<0.05), while self-reported quality of life improved in all of the evaluated patients (*p*<0.001). Nutritional support with hypercaloric, hyperproteic (with whey protein) OS, and vitamin D supplementation was associated with the maintenance of body composition and improvements in functionality and quality of life in patients with cancer undergoing systemic treatment.
Ngo-Huang et al. (19)	2023	USA	151	RCT	Pancreatic cancer	Aerobic and resistance exercise	Usual care (UC)	One hundred fifty-one patients were randomized. Objectively measured weekly activity (153.2 ± 135.6 and 159.8 ± 122.8 min in Arm A and B, respectively, *p* = 0.62) and self-reported weekly moderate-to-strenuous physical activity (107.4 ± 160.4 and 129.6 ± 161.6 min in Arm A and Arm B, respectively, *p* = 0.49) were similar, but weekly strength training sessions increased more in Arm B (by 1.8 ± 1.8 vs. 0.1 ± 2.4 sessions, *p* < 0.001). 6-min walk distance (6MWD) improved in both Arm A (mean change: 18.6 ± 56.8 m, *p* = 0.01) and Arm B (27.3 ± 68.1 m, *p* = 0.002). Quality of life and clinical outcomes did not differ significantly between the arms. Pooling patients in both study groups, exercise and physical activity were favorably associated with physical performance and clinical outcomes. Weekly objectively measured activity was favorably associated with changes in handgrip strength, self-reported physical functioning, and skeletal muscle index.
Ribeiro et al. (21)	2022	UK	15	RCT	Multiple types of cancers	Home-based resistance training (RT)/hospital-based resistance training	Standard care with information leaflet	We included 15 patients (53% men, with a median age of 68 years), 5 per arm. The home intervention had higher adherence (49% vs. 9% in hospital; *p* < 0.001). Acceptability was similar (93% in home and 95% in hospital; *p* = 0.179). No adverse events were recorded. Home-based RT can improve sarcopenia in advanced cancer.
Pring et al. (28)	2021	UK	58	RCT	Rectal cancer	Neuromuscular electrical stimulation plus standard care	Sham-neuromuscular electrical stimulation plus standard care	Preservation of muscle mass through early postoperative intervention with neuromuscular electrical stimulation (NMES) would allow a more rapid return to normal exercise and normal function, leading to greater muscle preservation and subsequently improved outcomes.
Tan et al. (13)	2021	China	232	RCT	Colorectal cancer	Dietary advice in combination with oral nutritional supplements (ONS)	Receive either dietary advice alone	The ONS group had a significantly lower sarcopenia prevalence (28.6% vs. 42.1%, *p* = 0.040).
Tumas et al. (14)	2020	Lithuania	70	RCT	Pancreatic cancer	Nutritional intervention with immunonutrition	Control	The rate of sarcopenia was lower in the intervention group than in the control group.
Van der Werf et al. (15)	2020	Netherlands	107	RCT	Colorectal cancer	Individualized nutritional counseling by a dietitian (NC)	Usual care (UC)	A total of 107 patients were enrolled (mean age, 65 years (SD, 11 years); 63% male). Mean change in skeletal muscle area from T0 until T1 was 2.5 (SD, 9.5) cm^2^, with no difference between the NC and UC groups (p ¼ 0.891). The proportion of patients with a clinically relevant decrease in skeletal muscle area of 6.0 cm^2^ did not differ (NC: 30% vs. UC: 31%, p ¼ 0.467). NC compared with UC had a significant positive effect on body weight (B coefficient 1.7, p ¼ 0.045), progression-free survival (p ¼ 0.039), and overall survival (p ¼ 0.046). NC of patients undergoing chemotherapy for metastatic colorectal cancer had no effect on muscle mass.
Moug et al. (22)	2020	UK	44	RCT	Rectal cancer	13–17 week telephone-guided graduated walking program	Standard care	Forty-four patients had a mean age of 66.8 years (SD: 9.6) and were male (64%), white (98%), American Society of Anesthesiologists class 2 (66%), co-morbid (58%), and overweight (72%) (body mass index: ≥25 kg/m^2^). At baseline, 14% were sarcopenic. At follow-up, 13 (65%) of patients in the prehabilitation group exhibited an increase in muscle mass, whereas 7 (35%) exhibited a decrease in muscle mass. Conversely, 16 (67%) controls experienced a decrease in muscle mass, and 8 (33%) showed an increase. An adjusted linear regression model estimated a mean treatment difference in the total psoas index of 40.2 mm^2^/m^2^ (95% CI − 3.4 to 83.7) between the groups in change from baseline (*p* = 0.07). Prehabilitation improved muscle mass in patients with rectal cancer who had neoadjuvant chemoradiotherapy (NACRT).
Ritch et al. (16)	2019	USA	61	RCT	Bladder cancer	Enriched oral nutrition supplement (ONS)	Multivitamin multimineral supplement (MVI)	The ONS group lost less weight (−5 kg vs. −6.5 kg, *p* = 0.04) compared to the MVI group. The proportion of patients with sarcopenia did not change in the ONS group but increased by 20% in the MVI group (*p* = 0.01). Mean length of stay (LOS) and 30-day hospital free days (HFDs) were similar between the groups. The ONS group had a lower rate of overall and major (Clavien grade ≥3) complications (48% vs. 67%; 19% vs. 25%, respectively) and lower readmission rates (17% vs. 7%), but the differences did not reach statistical significance. The proportion of patients with sarcopenia did not change in the ONS group but increased by 20% in the MVI group (*p* = 0.01).
Mazzuca et al. (17)	2019	Italy	47	RCT	Colorectal cancer	Whey protein	Placebo	Forty-seven patients were included in this preliminary analysis. Baseline characteristics were well-balanced between the two arms. During chemotherapy, 33 patients were reevaluated: anthropometric parameters (lean body mass from 68.5 to 71.2% vs. 68.7 to 66.3% and sarcopenia from 84 to 54% and 83 to 77% from baseline to T2 evaluation in arms A and B, respectively), nutritional status (mini nutritional assessment (MNA) > 24 = 100% [A] vs. 73.7% [B]), and toxicity (no adverse effects in 86% [A] vs. 29% [B] and 94% [A] vs. 29% [B] for hematological and gastrointestinal toxicities, respectively) resulted in being significantly different. In a univariate analysis, a condition of malnutrition risk according to MUST [relative risk (RR) = 7.5, *p* = 0.02] or MNA (RR = 1.45, *p* = 0.02) and ProLYOtin intake (RR = 0.12, *p* = 0.01) were found to be significantly predictive of chemotherapy toxicity. Sarcopenia decreased from 84 to 54% and from 83 to 77% from baseline to T2 evaluation in arms A and B, respectively.
Wright et al. (27)	2018	USA	28	RCT	Multiple types of cancers	Receive weekly injections of either 100 mg testosterone enanthate	Placebo	A total of 28 patients were enrolled, 22 patients were studied to completion, and 21 patients were included in the final analysis (12 placebos and nine testosterones). Adjunct testosterone increased lean body mass by 3.2% [95% confidence interval (CI), 0–7%], whereas those receiving placebo lost 3.3% (95% CI, 7 to 1%, *p* = 0.015). Although testosterone patients maintained more favorable body condition, sustained daily activity levels, and showed meaningful improvements in quality of life and physical performance, overall survival was similar in both treatment groups. Adjunct testosterone improved lean body mass compared with the placebo group.
Dawson et al. (23)	2018	USA	37	RCT	Prostate cancer	Resistance training	Control stretching	A total of 37 participants were randomized; 32 participated in the intervention (Exercise groups *n* = 13; non-exercise groups *n* = 19). At baseline, 43.8% of the participants were sarcopenic, and 40.6% met the criteria for metabolic syndrome (MetS). Post-intervention, EXE significantly improved lean mass (*d* = 0.9), sarcopenia prevalence (*d* = 0.8), body fat % (*d* = 1.1), strength (*d* = 0.8–3.0), and prostate cancer-specific quality of life (*d* = 0.9) compared to NoEXE (*p* < 0.05). No significant differences were observed between the groups for physical function or MetS-related variables except waist circumference (*d* = 0.8). A 12-week resistance training intervention effectively improved sarcopenia.
Dieli-Conwright et al. (20)	2020	USA	100	RCT	Breast cancer	Aerobic and resistance exercise	Usual care	Combined resistance and aerobic exercise effectively attenuated sarcopenia.
Kiwata et al. (29)	2017	USA	32	RCT	Prostate cancer	Resistance training and protein supplementation (RTPS)/RT/PS	Control	Resistance training and protein supplementation effectively improved sarcopenia.
Adams et al. (24)	2016	USA	200	RCT	Breast cancer	Resistance exercise training (RET)/aerobic exercise training (AET)	Usual care (UC)	RET consistently demonstrated superior effects to UC for improving sarcopenia-related outcomes.
Lee et al. (18)	2024	Korea	41	RCT	Pancreatic cancer	The case group consumed protein supplements containing 18 g of protein daily	The placebo group consumed a placebo with the same amount of carbohydrate	Protein supplementation improved sarcopenia.
Park et al. (25)	2023	Korea	30	RCT	Gastrointestinal cancers	A resistance and aerobic exercise program	Usual care	In the intervention group, muscle mass and physical functions were maintained.
Elnaggar et al. (26)	2025	Saudi Arabia/Egypt	62	RCT	Acute lymphoblastic leukemia	Adaptive variable-resistance training (adaptive-VRT)	Standard exercise protocol	Adaptive-VRT is a promising intervention for ameliorating chemotherapy-induced sarcopenia in pediatric acute lymphoblastic leukemia survivors.

**Table 2:** Efficacy comparison of CRS intervention categories.

Intervention category	Key outcome indicators	Strengths	Limitations
Nutritional intervention	Muscle mass change Serum albumin/prealbumin Nutritional risk reduction	High safety; applicable to all CRS stages; easy to implement.	Less effective in severe CRS (muscle loss >10%); dependent on patient absorption capacity.
Exercise intervention	Muscle strength (grip strength and knee extension) Physical function (6-minute walk test (6MWT) and Short Physical Performance Battery (SPPB) Muscle mass change	No adverse effects; enhances long-term functional resilience.	Poor adherence (30–40% dropout rate in elderly/advanced cancer patients); requires professional guidance.
Pharmacological intervention	Appetite improvement Muscle mass preservation inflammatory marker reduction	Rapid response in patients with severe cachexia.	Adverse effects (e.g., thromboembolism with megestrol; fluid retention with GH); limited data on long-term safety.
Multidisciplinary intervention	Composite outcomes (muscle mass + strength + QoL) Hospitalization Duration—complication rate	Integrates personalized nutrition, exercise, and medication; addresses multi-factorial pathogenesis.	Dependent on well-established MDT workflows, with resource-intensive, uneven implementation across healthcare settings.

**Table 3:** Risk of bias in studies.

Author	Randomization	Similar groups	Equal treatment	Equal analysis	Blinding
Herrera-Martínez et al. (12)	Yes	Yes	Yes	Yes	Unclear
Ngo-Huang et al. (19)	Yes	Yes	Yes	Yes	Unclear
Ribeiro et al. (21)	Yes	Yes	Yes	Yes	No
Pring et al. (28)	Yes	Yes	Yes	Yes	Yes
Tan et al. (13)	Yes	Yes	Yes	Yes	Unclear
Tumas et al. (14)	Yes	Yes	Yes	Yes	Yes
van der Werf et al. (15)	Yes	Yes	Yes	Yes	Unclear
Moug et al. (22)	Yes	Yes	Yes	Unclear	Unclear
Ritch et al. (16)	Yes	Yes	Yes	Yes	No
Mazzuca et al. (17)	Yes	Yes	Yes	Yes	Yes
Wright et al. (27)	Yes	Yes	Yes	Yes	Yes
Dawson et al. (23)	Yes	Yes	Unclear	Yes	Unclear
Dieli-Conwright et al. (20)	Yes	Yes	Yes	Yes	Yes
Kiwata et al. (29)	Yes	Yes	Yes	Yes	Unclear
Adams et al. (24)	Yes	Yes	Yes	Yes	Unclear
Lee et al. (18)	Yes	yes	Yes	Unclear	No
Park et al. (25)	Yes	Yes	Yes	Yes	Yes
Elnaggar et al. (26)	Yes	Yes	Yes	Yes	Unclear

### 3.3 Intervention strategies for cancer-related sarcopenia

**Figure 2:**
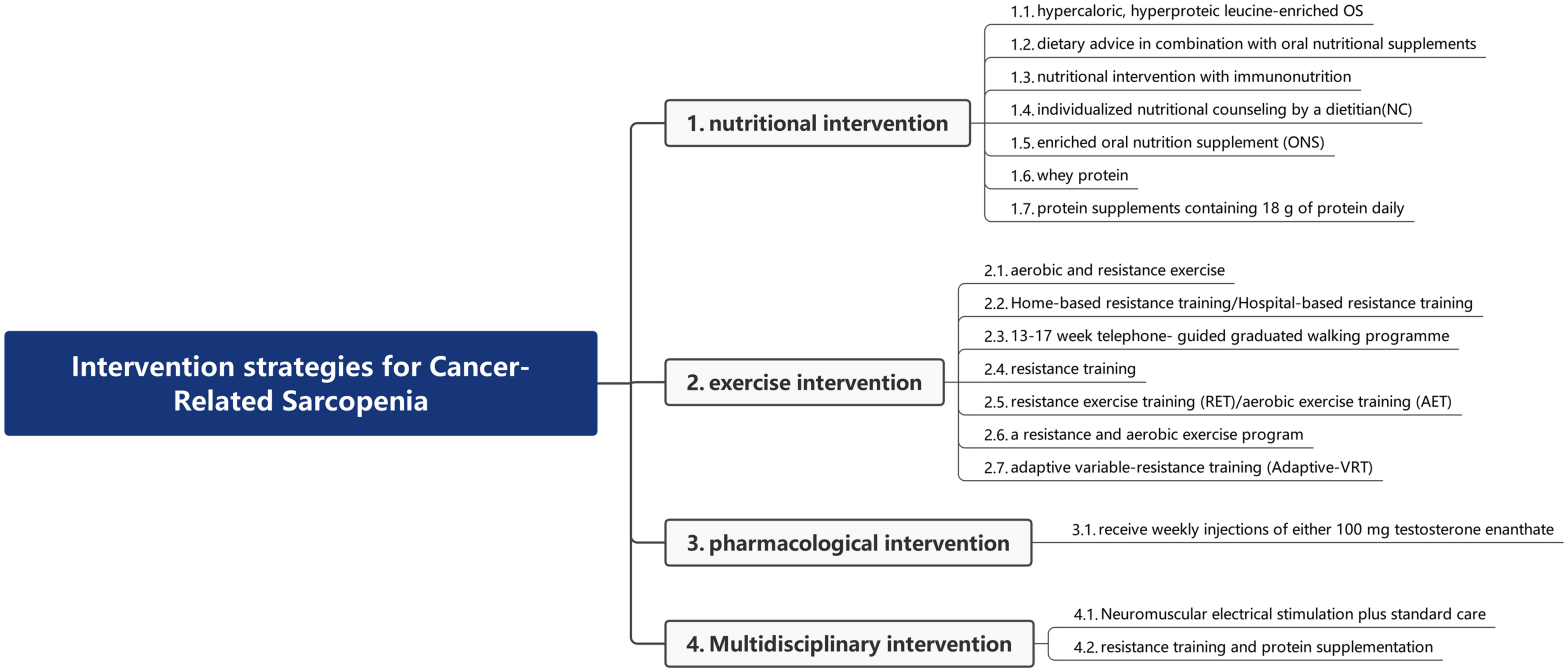
The results were analyzed by a content analysis. The interventions for CRS comprised four major categories: nutritional interventions, exercise interventions, pharmacological interventions, and multidisciplinary interventions.

In this review, we summarized interventions for CRS, including nutritional, exercise, pharmacological, and multidisciplinary approaches. The literature classification diagram is shown in Figure 2. In clinical studies of CRS, heterogeneity was prevalent, with core manifestations including differences in intervention effects and low comparability of data. This primarily stemmed from differences in the implementation of intervention methods, differences in the pathological characteristics of tumor types, and differences in the selection of outcome measurement indicators and tools.

#### 3.3.1 Nutritional intervention

This review included seven studies examining nutritional interventions for CRS. Herrera-Martínez et al. (12) reported that nutritional support with hypercaloric, hyperproteic OS (including whey protein), and vitamin D supplementation was associated with the maintenance of body composition. Additionally, dietary advice in combination with oral nutritional supplements (ONS) was linked to a significantly lower sarcopenia prevalence (13). The randomized controlled trial by Tumas et al. (14) provided evidence that nutritional intervention, specifically immunonutrition, was associated with a lower rate of sarcopenia in the intervention group than in the control group. In contrast, Van der Werf et al. (15) reported that individualized nutritional counseling (NC) by a dietitian for patients undergoing chemotherapy for metastatic colorectal cancer had no effect on muscle mass. Ritch et al. (16) reported that, with enriched oral nutrition supplements (ONS), the proportion of patients with sarcopenia did not change in the ONS group but increased by 20% in the control group (*p* = 0.01). Another randomized controlled trial demonstrated that whey protein supplementation significantly reduced the incidence of sarcopenia (17). Lee et al. (18) reported that the case group participants consumed protein supplements containing 18 g of protein daily, which led to improvements in sarcopenia.

#### 3.3.2 Exercise intervention

Among the studies included in this review, two reported that aerobic and resistance exercise were positively correlated with skeletal muscle index, effectively attenuating sarcopenia (19, 20). One study reported that home-based resistance training could improve sarcopenia in advanced cancer (21). Moug et al. (22) reported that a 13–17 week telephone-guided graduated walking program, prehabilitation, improved muscle mass in patients with rectal cancer. Dawson et al. (23) reported that a 12-week resistance training intervention effectively improved sarcopenia. The randomized controlled trial by Adams et al. (24) provided evidence that resistance exercise training consistently demonstrated superior effects to usual care for improving sarcopenia-related outcomes. Park et al. (25) reported that a resistance and aerobic exercise program significantly reduced the incidence of sarcopenia. Elnaggar et al. (26) indicated that adaptive-VRT is a promising intervention for ameliorating chemotherapy-induced sarcopenia in pediatric acute lymphoblastic leukemia survivors.

#### 3.3.3 Pharmacological intervention

One study reported that receiving weekly injections of either 100 mg of testosterone enanthate or adjunct testosterone improved lean body mass compared with the placebo group (27).

#### 3.3.4 Multidisciplinary intervention

Pring et al. (28) reported that neuromuscular electrical stimulation (NMES) combined with standard care helped to preserve muscle mass through early postoperative intervention, with NMES allowing a more rapid return to baseline. Another study reported that resistance training and protein supplementation effectively improved sarcopenia (29).

## 4 Discussion

As a multifactorial-driven complex syndrome, CRS requires intervention strategies that address muscle metabolic imbalance, the inflammatory microenvironment, and the superimposed effects of cancer therapy. In recent years, although some progress has been made in CRS intervention research, numerous challenges remain in clinical translation and individualized application.

Nutritional support and exercise training were regarded as the cornerstone interventions for CRS, yet the limitations of their isolated application were becoming increasingly evident. While high-protein diets could promote muscle protein synthesis, advanced cancer patients often exhibit reduced protein utilization due to metabolic disturbances (e.g., insulin resistance and elevated inflammatory cytokines). In such cases, combined resistance training enhances skeletal muscle sensitivity to amino acids by activating the mTOR pathway, thereby amplifying protein synthesis efficiency and creating a metabolic “synergistic effect.” For instance, a randomized controlled trial in prostate cancer patients demonstrated that resistance training combined with protein supplementation significantly improved sarcopenia (29). However, cancer patients frequently struggle to meet target nutritional intake due to anorexia, gastrointestinal dysfunction, or treatment-related side effects (e.g., chemotherapy-induced nausea). In such cases, personalized adjustments—such as enteral nutrition or ONS—are often required. Notably, while nutritional support alone may delay muscle loss, its impact on muscle strength and physical function remains limited; thus, dual optimization of “quality and quantity” necessitates integration with exercise interventions. Moreover, the modality and intensity of exercise must be dynamically tailored to patients’ functional status. For example, late-stage or severely debilitated patients may benefit from low-intensity progressive training (e.g., breathing exercises and anti-gravity movements). Early-stage patients could achieve superior outcomes with high-intensity interval training (HIIT) (30).

Pharmacological interventions for CRS remain exploratory, with several targeted approaches showing promise but facing clinical challenges. Androgen receptor modulators (e.g., enobosarm) and myostatin antibodies (e.g., bimagrumab) have demonstrated potential in reversing muscle atrophy by selectively targeting anabolic and catabolic pathways (31). However, the clinical application of anabolic agents (e.g., GH/IGF-1 axis modulators) is significantly constrained by their potential tumor-promoting effects, necessitating rigorous benefit–risk assessment—particularly in hormone-sensitive malignancies (e.g., breast and prostate cancers) (32). Anti-inflammatory agents mitigate muscle wasting by suppressing systemic inflammation, yet their long-term use is limited by gastrointestinal and cardiovascular toxicity. Consequently, future research must prioritize precision patient stratification and the development of tissue-selective targeted therapies.

The complexity of CRS necessitates transcending traditional unimodal interventions by establishing multidisciplinary team (MDT) models integrating oncology, nutrition, rehabilitation, and psychological care. For instance, the successful implementation of prehabilitation in colorectal cancer demonstrated that a 13–17 week preoperative rehabilitation program significantly improved muscle mass in patients with rectal cancer (22). During chemoradiotherapy, concurrent nutritional assessment and prehabilitation training effectively prevented muscle loss and enhanced treatment tolerance (33, 34). Psychological interventions demonstrated significant value in improving cancer-related fatigue (CRF) and treatment adherence, although they were frequently overlooked in clinical practice (35). Notably, implementing the MDT model required overcoming practical challenges such as unequal medical resource allocation and inefficient interdisciplinary communication, which was particularly pronounced in primary care institutions, thereby necessitating optimization through standardized protocols and telemedicine technologies.

The management of CRS relies on multidimensional interventions, including nutrition, exercise, and pharmacology. However, single or short-term interventions fail to address patients’ full-course needs, and long-term follow-up is key for improving prognosis. Meanwhile, clinical implementation also faces multiple practical barriers, such as inadequate MDT mechanisms, patient compliance issues and individual differences, a lack of unified long-term assessment standards and tools, and insufficient allocation of medical resources coupled with inadequate primary care capabilities.

## 5 Conclusion

The management of CRS should follow a “primary prevention-multidimensional intervention-long-term management” framework, integrating nutrition, exercise, pharmacotherapy, and MDT models to optimize functional outcomes and quality of life. Future research must overcome precision and translational medicine barriers while addressing cost-effectiveness and accessibility to benefit the broader patient population.
